# Calmodulin Directly Interacts with the Cx43 Carboxyl-Terminus and Cytoplasmic Loop Containing Three ODDD-Linked Mutants (M147T, R148Q, and T154A) that Retain α-Helical Structure, but Exhibit Loss-of-Function and Cellular Trafficking Defects

**DOI:** 10.3390/biom10101452

**Published:** 2020-10-17

**Authors:** Li Zheng, Sylvie Chenavas, Fabien Kieken, Andrew Trease, Sarah Brownell, Asokan Anbanandam, Paul L. Sorgen, Gaelle Spagnol

**Affiliations:** 1 Department of Biochemistry and Molecular Biology, University of Nebraska Medical Center, Omaha, NE 68198, USA; li.zheng@unmc.edu (L.Z.); sylvie.chenavas@hotmail.fr (S.C.); fabienkieken@gmail.com (F.K.); andrew.trease@unmc.edu (A.T.); sebrownell@gmail.com (S.B.); 2 Biomolecular NMR Core Facility, University of Kansas, Lawrence, KS 66045, USA; asokan.ph.d@gmail.com

**Keywords:** gap junctions, connexin43, cytoplasmic loop domain, ODDD, calmodulin, NMR, circular dichroism

## Abstract

The autosomal-dominant pleiotropic disorder called oculodentodigital dysplasia (ODDD) is caused by mutations in the gap junction protein Cx43. Of the 73 mutations identified to date, over one-third are localized in the cytoplasmic loop (Cx43CL) domain. Here, we determined the mechanism by which three ODDD mutations (M147T, R148Q, and T154A), all of which localize within the predicted 1-5-10 calmodulin-binding motif of the Cx43CL, manifest the disease. Nuclear magnetic resonance (NMR) and circular dichroism revealed that the three ODDD mutations had little-to-no effect on the ability of the Cx43CL to form α-helical structure as well as bind calmodulin. Combination of microscopy and a dye-transfer assay uncovered these mutations increased the intracellular level of Cx43 and those that trafficked to the plasma membrane did not form functional channels. NMR also identify that CaM can directly interact with the Cx43CT domain. The Cx43CT residues involved in the CaM interaction overlap with tyrosines phosphorylated by Pyk2 and Src. In vitro and in cyto data provide evidence that the importance of the CaM interaction with the Cx43CT may lie in restricting Pyk2 and Src phosphorylation, and their subsequent downstream effects.

## 1. Introduction

Gap junctions are integral membrane proteins that enable direct cytoplasmic exchange of ions and low molecular-mass molecules between adjacent cells [1]. They also provide a pathway for propagating and amplifying intercellular signal transduction cascades triggered by cytokines, growth factors, and other molecules involved in development, growth, and differentiation. Dysfunctional gap junction intercellular communication causes a number of human pathologies and genetic diseases [2]. Gap junctions are formed by the apposition of connexons from adjacent cells. Each connexon is a hexamer of connexin proteins. Though the 21 human connexin isoforms share significant sequence homology, there is major divergence in the primary sequence of the cytoplasmic domains, particularly the cytoplasmic loop (CL) and carboxyl-terminus (CT).

The CT plays a role in the trafficking, localization, and turnover of gap junction channels via numerous post-translational modifications and protein–protein interactions [3,4,5,6,7]. The CT is also important for regulating junctional conductance and voltage sensitivity [8,9,10,11]. We and others have shown that the CT binds multiple proteins (e.g., >25 known for connexin43 (Cx43)), some of which have been shown to modulate channel function (for review, see [12,13]). Conversely, only a few protein partners have been identified to directly bind the CL. For example, in the context of pH-gating regulation due to ischemia, we previously identified in vitro a pH-dependent interaction between a peptide corresponding to the second half of the Cx43CL (the “L2” peptide, D119-K144) and the Cx43CT domain [14]. Structural analysis showed that at pH 5.8 the L2 peptide contains two short *α*-helices (each with a His residue; H126 and H144) separated by an intrinsically disordered region, and its affinity for the CT is greater than at pH 7.4 [14]. Single channel analysis revealed that the same peptide, when presented in the patch pipette, interfered with the occurrence of the residual state and prolonged open time [15]. A similar result was observed with Cx43 H144E gap junction channels; a mutation that disrupted the secondary structure of the L2 and interaction with the CT [16]. These results led to the suggestion that Cx43 pH-regulation involves the intramolecular interaction between the L2 region of CL (“receptor”) and the CT domain (“gating particle”).

The Ca^2+^ binding protein calmodulin (CaM) is another binding partner of the Cx43 (and other connexin isoforms) CL domain [17,18,19,20]. In general, increase in intracellular Ca^2+^ concentration leads to a CaM-connexin interaction causing gated closure of gap junction channels [19,21,22,23,24,25,26,27,28]. CaM was shown to directly interact with a Cx43CL peptide (residues K136-S158) that contains a predicted 1-5-10 hydrophobic CaM-binding motif (residues M147, L151, and I156; [17]). This causes CaM to fully collapse around the Cx43CL peptide in a “classical” mode of binding and the Cx43CL to form α-helical structure [17,29]. Intracellular addition of this Cx43CL peptide in a patch clamp experiment prevented the Ca^2+^ induced decline in gap junctional conductance [25]. Also located within these Cx43CL residues are mutations (e.g., G138D/R/S, G143S/D, K144E, V145G, M147T, R148Q/G, and T154A/N) linked to the autosomal-dominant human disorder named oculodentodigital dysplasia (ODDD; for review, see [30,31]). This syndrome is associated with abnormalities affecting the eyes, dentition, and digits of the hands and feet [32,33,34]. Neurologic symptoms are also frequently described [35] as well as a few cases of increased susceptibility to spontaneous arrhythmias [36,37]. Of those ODDD mutations characterized for the mechanism of gap junction dysregulation within the Cx43CL domain that binds CaM (residues K136-S158), G138R, G143S, and T154A increased hemichannel function and/or decreased gap junction channel function [37,38,39,40].

In this report, our goal was to structurally characterize the full length Cx43CL domain (M100-Y155) and confirmed the predicted 1-5-10 CaM-binding motif using a series of Cx43CL mutants. We also determined that three ODDD mutations (M147T, R148Q, and T154A; [41,42,43]) directly localized within the predicted 1-5-10 CaM-binding motif had little-to-no effect on the ability of the Cx43CL domain to form an α-helical structure and bind CaM. However, these mutations did decrease gap junction intercellular communication through a combination of limiting plasma membrane localization and forming non-functional channels. Additionally, we discovered that in addition to binding to the CL domain, CaM can also directly interact with the Cx43CT. The importance of the CaM interaction with the Cx43CT may lie in restricting Pyk2 and Src phosphorylation, and their subsequent downstream effects.

## 2. Materials and Methods

### 2.1. Expression and Purification of Recombinant Proteins, and Peptide Synthesis

The rat Cx43CL_100-155_, Cx43CL_100-158_, Cx43CT_236-382_, and Cx43CT_262-327_ polypeptides (unlabeled, ^15^N- or ^15^N^13^C-labeled) cloned into the bacterial expression vector pGEX-6P-2 (GST-tagged; Amersham Biosciences) were expressed and purified as previously described [14]. Cx43CL_100-158_ and Cx43CT_262-327_ were obtained by adding or deleting residues in the Cx43CL_100-155_ and Cx43CT_236-382_ plasmids respectively, using the Quick Change Lightning kit (Agilent, #210518, Santa Clara, CA, USA). Cx43CL_100-158_ ODDD mutants M147T, R148Q, T154A polypeptides, and Cx43CT_236-382_ S255,279,282D and Y265,313D were obtain by introducing the specific point mutation in the Cx43CL or Cx43CT plasmids, respectively, using the same kit. Purity and analysis for degradation were assessed for each polypeptide by SDS-PAGE. Cx43CL_136-158_ peptides described in Figure 5c were synthesized by LifeTein (95% purity). CaM was expressed (cloned in pAED4 vector; unlabeled or ^15^N-labeled) and purified following the previously described method in [44]. All polypeptides were equilibrated in 1x phosphate-buffered saline (1x PBS) at pH 5.8 or KCl 100 mM, CaCl_2_ 5 mM at pH 6.5 using NAP-5 columns (GE Healthcare, #17-0853-01, Chicago, MI, USA) by dialysis in Slide-A-Lyzer G2 Dialysis Cassettes, 2K MWCO (Thermo Scientific, #87717, Waltham, MA, USA).

### 2.2. Nuclear Magnetic Resonance (NMR)

**Figure 1:**
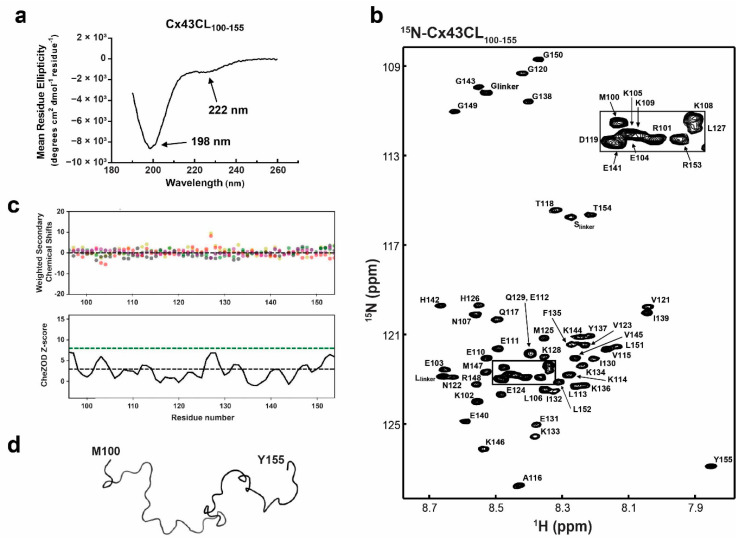
Structural analysis of the Cx43CL_100-155_ domain. (**a**) Circular dichroism (CD) spectrum of Cx43CL_100-155_ in 1× PBS at pH 5.8. Peaks at 198 and 222 nm, typical of random coiled and α-helix containing proteins, respectively, have been labeled. (**b**) ^15^N-HSQC spectrum of ^15^N-labeled Cx43CL_100-155_ in 1× PBS (pH 5.8, 7 °C). Each residue (but proline) has been labeled. (**c**) Top panel: weighted difference between observed and predicted shifts, shown with salmon, grey, green, beige, and purple dots for Cα, Cβ, Hα, Hβ, and N respectively. Bottom panel: Z score represented as a black line drawn from the score of each residues. Threshold lines for totally disordered Z < 3 (black dashes) and ordered Z > 8 (green dashes) are represented. (**d**) Backbone trace of the lowest energy Cx43CL_100-155_ NMR structure.

NMR data were acquired at 7 °C or 25 °C using a 600 MHz Varian INOVA (Agilent Technologies, Santa Clara, CA, USA) or a 600 MHz Bruker Avance-III HD spectrometer (Bruker, Billerica, MA, USA) outfitted with a cryoprobe at the NMR Facility of the University of Nebraska Medical Center. NMR data shown in the Supplementary Figure S1 were collected at 25 °C using a 800 MHz Bruker Avance NMR instrument (Bruker, Billerica, MA, USA) fitted with a TCI cryoprobe at the Biomolecular NMR Laboratory of the University of Kansas. Backbone sequential assignments for the Cx43CL_100-155_ (Figure 1b) and CaM in presence of Cx43CL_136-158_ peptide (Supplementary Figure S1) were obtained using experiments previously described [14,45]. NMR spectra were processed using NMRPipe (IBBR, Rockville, MD, USA) [46] and analyzed using NMRView (https://nmrfx.org) [47].

### 2.3. Structure Calculation

Model structures were calculated by simulated annealing using torsion angle dynamics as implemented in the program Crystallography & NMR System (http://cns-online.org/v1.3/) [48]. NOE cross-peaks classified as strong, medium, and weak were converted into distance restraints of 1.8–2.5 Å, 1.8–3.5 Å, and 1.8–5.5 Å, respectively. The 10 best energy minimized structures were evaluated using PROCHECK-NMR (EMBL-EBI, Cambridge, UK) [49].

### 2.4. Calculating Binding Affinity by NMR

Gradient-enhanced two-dimensional ^15^N-HSQC experiments were collected to obtain binding isotherms of either the ^15^N-labeled CaM or Cx43CT_236-382_ domain at a constant concentration (30–100 μM) in the absence or presence of various amounts (7.5 to 400 μM) of different unlabeled ligands: Cx43CL_136-158_ peptides, Cx43CT_262-327_, Cx43CT_236-382_ wild type (WT), and MAPK and Src phosphomimetic mutants. Data were acquired with 1024 complex points in the direct dimension and 128 complex points in the indirect dimension. Chemical shift variations were calculated according to the formula Δσ = √((Δδ_HN_)^2^ + (Δδ_N/5_)^2^) and plotted as a function of ligand concentration. Dissociation constants (K_D_) were calculated by nonlinear fitting of the titration curves using GraphPad Prism 7.0 (GraphPad Software, La Jolla, CA, USA) and averaging at least six curves. The same formula was used to estimate the change in chemical shift of the Cx43CL_100-155_ residues because of the effect of trifluoroethanol (TFE; Acros Organic, #139750250, Thermo Scientific, Waltham, MA, USA).

### 2.5. Circular Dichroism (CD)

CD experiments were performed on a JASCO J-815 CD spectrometer at 7 °C in the far UV (260–190 nm) with a 0.1 mm path length quartz cell, using a bandwidth of 1 nm, an integration time of 1 s, and a scan rate of 50 nm/min. Each spectrum is the average of 5 scans. All spectra were corrected by subtracting the solvent spectrum acquired under identical conditions. The protein concentration for each sample was 200–500 μM in 1× PBS at pH 5.8. For CD data collection in presence of TFE, 10× PBS was used to keep the final sample at a constant buffer concentration corresponding to 1x PBS. All CD data were processed using the Spectra Analysis software of Jasco Manager Version 2 (Jasco, Easton, PA, USA).

### 2.6. Cell Culture

Parental HeLa cell line was cultured at 37 °C in a humidified 5% CO_2_ atmosphere incubator, in Dulbecco’s modified Eagle medium (Hyclone, #SH30243.01, Cytiva, Marlborough, MA, USA) supplemented with 10% fetal bovine serum (Atlanta Biologicals, #S11150, R&D Systems, Minneapolis, MN, USA), 2 mM L-glutamine (HyClone, SH30034.01), 1% pen-strep (Corning, #30-002-CL, Corning, NY, USA), and 0.2% Normocin (Invivogen, #ant-nr-2, San Diego, CA, USA). HeLa cell lines stably expressing Cx43 WT (HeLa^Cx43^) or ODDD mutations M147T, R148Q, or T154A were generated as previously described [50]. Briefly, 80% confluent parental HeLa cells were transfected with Cx43 WT or ODDD mutations containing pD2529 (puromycin resistant) plasmid using Lipofectamine 2000 (Invitrogen, # 11668019) at a 1:1 (reagent:plasmid) ratio in OptiMem (Life Technologies, #31985070, Carlsbad, CA, USA) under antibiotic free conditions. After re-plating and once colonies were formed, Western blot and immunofluorescence were used to screen the clones (α-Cx43, Sigma #6219, 1:10,000, MilliporeSigma, St. Louis, MO, USA).

### 2.7. Antibodies

Detection reagents, manufacturers, and concentration ranges for all primary and secondary antibodies utilized in Western blot imaging are as follow: Pyk2 (Cell Signaling #3480S, 1:1,000, Danvers, USA), Pyk2 pY402 (Abcam #4800, 1:1,000, Cambridge, UK), PKCα (Cell Signaling #2056S, 1:1,000), PKCα pT638 (Abcam #32502, 1:5,000), Src (Cell Signaling #2123S, 1:1,000), Src pY416 (Cell Signaling #6943, 1:1,000), ERK1/2 (Cell Signaling #4695, 1:2,000), ERK1/2 pT202/pY204 (Cell Signaling # 9101S, 1:2,000), Cx43 pY265 (Abcam #193373, 1:1,000), Cx43 pS279 (Invitrogen # PA5-64640, 1:2,000), Cx43 pS282 (Invitrogen # PA5-64641, 1:2,000), Cx43 (Sigma #6219, 1:10,000), Anti-rabbit IgG - HRP-linked Antibody (Cell Signaling #7074, 1:1,000 to 10,000), Anti-mouse IgG - HRP-linked Antibody (Cell Signaling #7076, 1:1,000).

### 2.8. Immunofluorescence

After reaching 100% confluency, cells seeded on glass coverslips were fixed in 4% paraformaldehyde (1× PBS, pH 7.4) and then blocked in 5% goat serum (1× TBS, 0.4% Triton X-100). Coverslips were then incubated with the appropriate primary antibody (α-Cx43, Sigma #6219, 1:200) diluted in blocking buffer overnight at 4 °C. Fluorescent secondary antibody (α-Rb Alexa Fluor 488, Cell Signaling #4412, 1:300) was then applied, followed by DAPI (100 ng/mL in 1× TBS). Coverslips were mounted on a drop of SlowFade anti-fade (Life Technologies, Carlsbad, CA, USA # S36972), sealed with clear nail polish, and imaged.

### 2.9. In Situ Detergent Extraction

The in situ detergent extraction was previously described [51]. Briefly, HeLa cells seeded on glass coverslips (Neuvitro, Vancouver, BC, Canada) and cultured to ~90% confluency were incubated with gentle agitation in 1% Triton X-100 extraction buffer (1% Triton X-100, 1× complete protease inhibitor cocktail (Roche #11836145001, Basel, Switzeland) + EDTA, 2 mM PMSF, 2 μM Pepstatin in 1× PBS). Following extraction, the cells were fixed, immunostained, mounted, and imaged.

### 2.10. Scrape Loading Dye Transfer Assay

Cell communication was evaluated as previously described [52]. Briefly, stably transfected HeLa cells described above were grown to 100% confluency. Media was replaced with pre-warmed dye transfer solution (0.25% Lucifer yellow CH (Life Technologies # L453) and 0.1% Texas Red 1 × 10^4^ (Life Tech # D1863) in 1× PBS at 37 °C) and cells were immediately scrape-loaded with a fine edged micro-scalpel by three longitudinal scratches. After incubating at 37 °C for 4 min, cells were rinsed with 1× PBS and further incubated in antibiotic free media for 6 min. Dye spread was then stopped by washing the cells with 1× PBS containing 1 mM CaCl_2_ and 1 mM MgCl_2_. Cells were finally fixed, DAPI stained, mounted, and imaged.

### 2.11. Confocal Imaging

Confocal microscopy images were obtained using a Zeiss LSM 800 Confocal system (Zeiss, Oberkochen, Germany) at the Advanced Microscopy Core Facility of the University of Nebraska Medical Center. Numerical aperture objectives, excitation, and filter sets were carefully selected according to the nature of the fluorescence.

### 2.12. In Vitro Kinase Assay

Purified GST-tagged Cx43CT_236-382_ was incubated in presence of either Src, ERK1, or ERK2 kinase in the appropriate buffer according to manufacturer protocol (SignalChem, #S19-18G, M29-10G, M28-10G respectively, Richmond, VA, USA), and with or without CaM (90% saturation of the Cx43CT according to the K_D_ of the interaction). After incubation of 4 h at RT for Src, 6 h at 30 °C for ERK1, and 6 h at RT for ERK2, phosphorylation levels of the Cx43CT were detected by Western blot using Cx43 specific anti-phospho antibodies. Three independent experiments were performed and phosphorylation levels were quantified using the iBright Analysis Software and statistical analysis were performed in GraphPad Prism 8.0 using a Student’s t-test. *p*-values < 0.05 were considered statistically significant.

### 2.13. Ionomycin Treatment and Detergent Solubility Assay

After reaching 100% confluency, HeLa^Cx43^ cells seeded on 10-cm dishes were incubated in presence of 1 μM ionomycin (Sigma #I0634) for 2 min for equilibration and then either treated with 1 μM ionomycin only as control, or 1 μM ionomycin plus 20 mM Ca^2+^, 100 nM PMA (Sigma, # P1585), or 20 mM Ca^2+^/100 nM PMA and incubated for 30 min.

The Triton X-100 solubility assay was modified from a method described previously [53]. Briefly, lysate of HeLa^Cx43^ cells grown on 10-cm dishes was prepared by harvesting the cells in 1 mL of 1x PBS supplemented with Halt™ Protease Inhibitor Cocktail (Thermo Scientific #78429), pepstatin A, and PMSF, and briefly sonicated (2 × 10 sec). Protein lysate content was quantified by the BCA assay and normalized. Total of 450 μL of cell lysate was then reserved as total lysate while Triton X-100 was added to another 450 μL at a final concentration of 1% and incubated at 4 °C for 60 min with agitation. The Triton X-100 lysate was then separated into non-junctional (supernatant; soluble) and junctional (pellet; insoluble) fractions by ultra-centrifugation at 1 × 10^5^× *g* for 1 h at 4 °C. The pellet was solubilized in 450 μL of solubilization buffer (1× PBS, 8 M urea, 2.5% SDS, 0.1 M DTT, 1× Roche Complete + EDTA, 2 mM PMSF, and 2 μM Pepstatin A). After analysis of the insoluble fraction by Western blot, quantification from three independent replicates was measured using the iBright Analysis Software (version 3.1.3, Thermo Scientific) and statistical analysis was performed in GraphPad Prism 8.0 (San Diego, CA, USA) using the Ordinary one way Anova calculation. *p*-values < 0.05 were considered statistically significant.

## 3. Results

### 3.1. Structural Analysis of Cx43CL Residues M100-Y155

A well-accepted regulation mechanism for Cx43 relies on the pH-dependent interaction between the Cx43CT and Cx43CL [9,14,54]. Our laboratory showed by NMR that a Cx43 peptide corresponding to CL residues D119-K144 (L2 peptide) interacted with the Cx43CT domain at pH 5.8 [14]. The interaction was validated using the full-length Cx43CL (residues M100–Y155 (Cx43CL_100-155_); according to various TM prediction tools, TM2 stops anywhere between residues 98–102 and TM3 starts at 151–155; [55]), however, the structure of this longer Cx43CL construct remains unknown. To investigate the global folding of Cx43CL_100-155_ at pH 5.8, we used CD spectroscopy in the far-UV region (Figure 1a). The Cx43CL presented a major absorption at 198 nm, typical of a random coiled protein. Also observed was a weak but noticeable absorption at 222 nm. A pronounced double minimum at ~222 nm and ~208 nm, with the 222 nm peak being deeper than the 208 nm, is typical for an α-helical structure. In this case, the weak absorption at 222 nm suggested that some residues are sampling α-helical structure (i.e., interconverting between random coil and α-helix). Deconvolution tools available to calculate the percentage of secondary structure from CD spectra could not be used as they depend on databases derived from well-folded globular soluble proteins. Thereby they do not provide reliable estimates of secondary structure for peptides and predominantly random coiled proteins [56]. However, by comparing previous data collected in our laboratory, we estimated the α-helical content to be less than 5% [57].

Toward solving the Cx43CL_100-155_ solution structure and being able to map the binding site of protein partners, all ^1^H, ^15^N, ^13^C backbone resonances and most side chain resonances in 1x PBS at 7 °C and pH 5.8 were assigned. The chemical shift data of Cx43CL_100-155_ were deposited into the BioMag ResBank database, accession number 50201. The ^15^N-HSQC spectrum and assignment of the Cx43CL_100-155_ are displayed in Figure 1b. The ^15^N-HSQC is a two-dimensional NMR experiment where each amino acid (except proline) gives two correlated signals (or chemical shifts) that corresponds to its N-H amide group. The narrow proton chemical shift dispersion in the ^15^N-HSQC spectrum is consistent and in agreement with the CD data that showed the Cx43CL as being predominantly disordered. The absence of significant order was moreover confirmed by calculating the chemical shift Z-score (CheZOD Z-score) based on the global deviations from random coil chemical shifts (Figure 1c) [58,59]. The order/disorder content of the Cx43CL was assessed and while a Z-score at or above 8 is typical for a structured residue, a majority of the residues displayed a Z-score below 3, consistent with a mostly disordered domain. Interestingly, one segment (H126–Q129) did comprise values between 3 and 8 and this correlates with the structured region identified from the Cx43CL D119-K144 peptide (N122-L127; [14]). Unfortunately, NOE restraints used for the structure calculation were deficient in the medium- and long-range NOE connectivities needed for secondary and tertiary structure (data not shown). As expected, the structure calculation by torsion angle dynamics followed with refinement by simulated annealing, and energy minimization led to a family of structures that were intrinsically disordered (Figure 1d; backbone trace of the lowest energy “structure”). The very small amount of α-helix detected by CD (Figure 1a) and suggestive from the CheZOD Z-score were not identified by NMR. The most likely explanation is that the α-helical NOEs did not rise to the level of detection, since the NOE is a weighted average of the population and the α-helical population is low.

### 3.2. Propensity of the Cx43CL_100-155_ to Form α-Helical Structure

**Figure 2:**
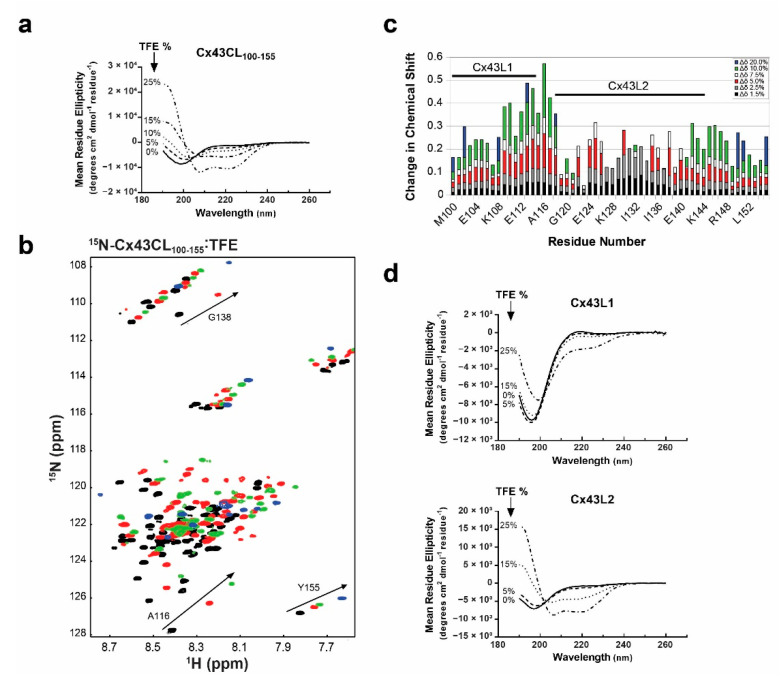
Propensity of the Cx43CL_100-155_ domain to form α-helical structure. (**a**) CD spectra of Cx43CL_100-155_ in the presence of increasing amount of trifluoroethanol (TFE) (percentages indicated on the left side of the curves). (**b**) ^15^N-HSQC spectrum of ^15^N-labeled Cx43CL_100-155_ only (black) has been overlaid with spectra obtained when increasing amount of TFE were added (5%, red; 10%, green; and 20%, blue). Spectra for the Cx43CL_100-155_ in the presence of 1.5%, 2.5%, and 7.5% of TFE were also collected and used for the measurement of the change in chemical shifts (panel **c**) but not shown because of the overlap of the peaks. (**c**) Each Cx43CL_100-155_ residue was plotted against their change in chemical shift as a function of TFE concentration. The two major affected areas have been labeled and a representative residue for each area is displayed in panel **b** (A116 for Cx43L1, G138 for Cx43L2, and Y155 for the area outside of L1 and L2). (**d**) CD spectra of Cx43L1 residues M100-A116 (top panel) and Cx43L2 residues D119-K144 (bottom panel) were collected in 1x PBS at pH 5.8 in the absence and presence of varying concentrations of TFE (percentages displayed in each panel).

Previously, we showed that the solution structure of the Cx43CL peptide D119-K144 contained two short α-helices [14], a result not observed in the final conformations of the full-length Cx43CL (residues M100-Y155). The additional 30 residues might render the construct more flexible and lead to a destabilization of the α-helices especially when in solution and not tethered to the TM2 and TM3. Therefore, we used trifluoroethanol (TFE), known to stabilize secondary protein structure and in particular α-helical configuration, to probe the helical propensity of this longer construct [60]. We initially collected CD spectra of the Cx43CL_100-155_ in the presence of various concentrations of TFE (Figure 2a). The addition of TFE from 5% to 25% caused the absorption minima to shift from 198 nm to 208 nm and 222 nm, consistent with the Cx43CL domain being able to form α-helical structure.

We then collected ^15^N-HSQC spectra of the ^15^N-Cx43CL_100-155_ in the presence of various concentrations of the co-solvent to gain a better understanding of the per-residue propensity to form α-helical structure (Figure 2b). These NMR signals are sensitive to the chemical environment and even small changes in structure and/or dynamics can change the chemical shift of an amino acid. As a result of the addition of TFE from 1.5% to 20%, two unique regions were identified when each amino acid was plotted against their changes in chemical shift (Figure 2c). One area had a cluster of residues which shifted the greatest in the presence of TFE (M100-A116, “L1”) and one area had a cluster of residues which shifted and then broaden beyond detection (D119-K144, “L2”). Synthetic peptides corresponding to each of these regions were generated and analyzed by CD to determine which Cx43CL domain has the highest propensity of forming an α-helix.

CD data were collected for each peptide in the absence and presence of various concentrations of TFE (Figure 2d). Peptide Cx43L1 was able to form some α-helical content with increased amount of TFE. However, at 25% of TFE, the spectrum was still typical of a predominantly random coiled protein. On the other hand, Cx43L2 peptide clearly adopted an α-helical structure. Interestingly, the longest stretch of residues with a Z-score close to 8 in the CheZOD Z score calculation encompasses residues contained in the Cx43L2 peptide. These results confirm that the Cx43CL_100-155_ has a strong propensity to form α-helical structure and that the L2 region is largely responsible for this propensity.

### 3.3. Structural Analysis of Cx43CL Residues M100-S158

**Figure 3:**
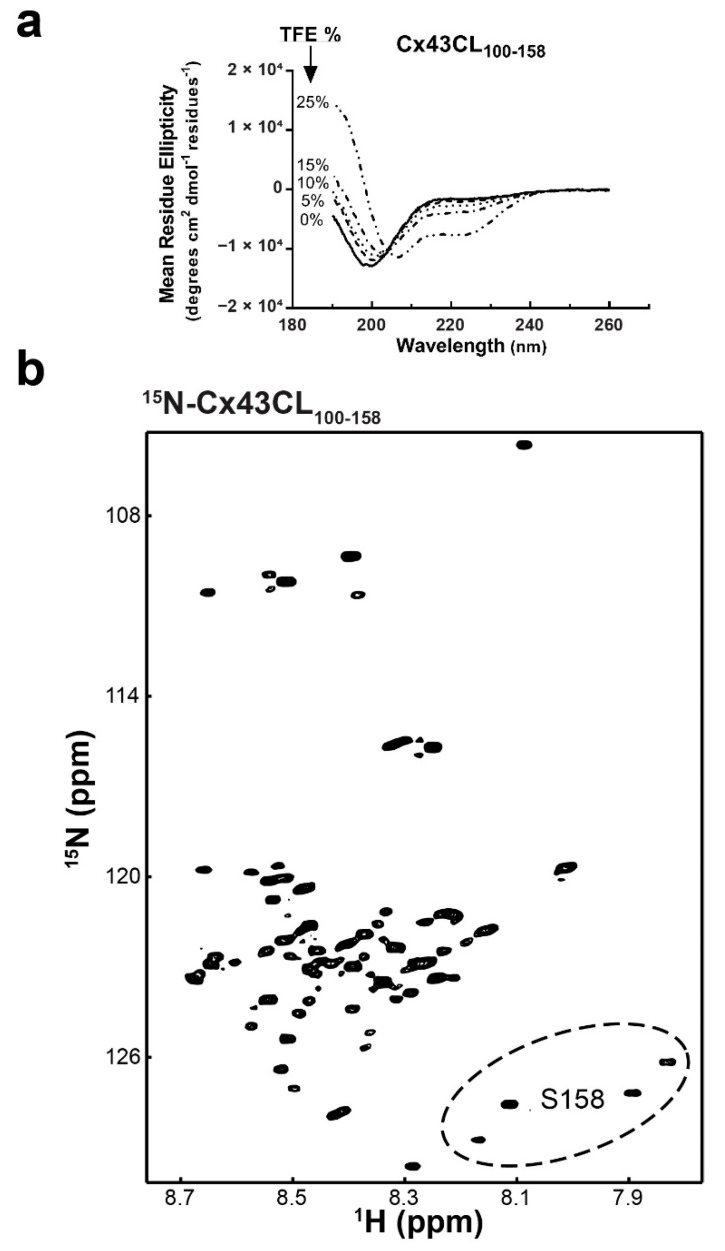
Structural analysis of the Cx43CL_100-158_ domain. (**a**) CD spectra of Cx43CL_100-158_ in the presence of increasing amount of TFE (percentages indicated on the left side of the curves). (**b**) ^15^N-HSQC spectrum of ^15^N-labeled Cx43CL_100-158_ in 1× PBS (pH 5.8, 7 °C). Example of the CT region sampling multiple confirmations is shown with residue S158 peaks (circled region).

After initiation of the Cx43CL M100-Y155 structural study, Zhou et al. [17] identified that the CaM binding domain for the Cx43CL involves residues K136-S158. Therefore, we extended our work to examine a longer Cx43CL construct containing residues M100-S158 (Cx43CL_100-158_). CD spectroscopy of Cx43CL_100-158_ presented a major absorption at 198 nm, similar to Cx43CL_100-155_, however the dip was more pronounced (Figure 3a vs. Figure 2a). In addition, TFE from 5 to 25% for Cx43CL_100-158_ did not elicit the same degree of α-helical content as Cx43CL_100-155_ (absorbance 208 nm >> 222 nm). The ^15^N-HSQC of the Cx43CL_100-158_ helped explain why this construct had reduced ability to form α-helical structure (Figure 3b). In the Cx43CL_100-158_ spectrum, 59 peaks should be observed; yet the spectrum contained at least 84 peaks. This is the result of a number of C-terminal residues sampling multiple different confirmations (e.g., see Figure 3b, residue S158–circled region), which is not conducive for α-helical structure. These observations inhibited any further structural determination of Cx43CL_100-158_ by NMR.

### 3.4. Structural Analysis of Cx43CL_100-158_ ODDD Mutants

**Figure 4:**
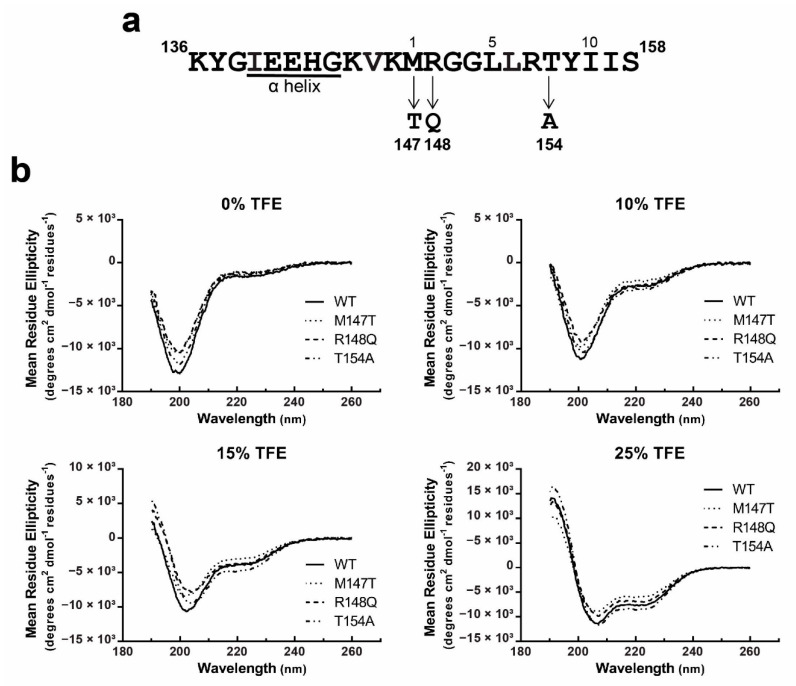
Structural analysis of the Cx43CL ODDD mutants M147T, R148Q, and T154A. (**a**) ODDD mutations localized in the predicted 1-5-10 CaM binding domain of Cx43CL are represented. (**b**) α-helical content of the Cx43CL_100-158_ ODDD mutants M147T (dotted line), R148Q (dash line), and T154A (dotted and dash line) was estimated and compared with the Cx43CL_100-158_ WT (black line) by collecting CD spectra in the presence of 0%, 10%, 15%, and 25% TFE.

Gap junctions are subject to regulation by intracellular Ca^2+^, in part, by an interaction between CaM and the Cx43CL (for review, see [28]). Upon binding, CaM induces an α-helical structure in a Cx43CL peptide containing residues A136-S158 [17]. This Cx43CL region contains a predicted 1-5-10 hydrophobic residue motif (M147-L151-I156) consistent with other well characterized CaM-targeting proteins [61]. Also located between Cx43CL residues M147-I156 are a subset of mutations that cause ODDD (M147T, R148Q, and T154A; Figure 4a) [41,42,43]. To assess the impact of these mutations within the Cx43 CaM binding site, we first investigated if a change in the overall structure (or propensity to form structure) could be a mechanism responsible for the various phenotypes observed in ODDD patients. We collected CD spectra for each of the full length Cx43CL_100-158_ mutants in the absence or presence of TFE (Figure 4b). Without TFE, CD spectra all showed a similar pattern as the Cx43CL WT with a major absorption around 198 nm, typical of a random coiled protein. All of them also presented a weak absorption at 222 nm demonstrating the possibility that some residues are sampling an α-helical conformation. With increasing concentrations of TFE (10% to 25%), each of the Cx43CL ODDD mutants had a similar amount of α-helical structure as the WT. Thus the data suggest that the ODDD mutations do not alter the propensity of the Cx43CL domain to form secondary structure.

### 3.5. Functional Implications of the Cx43CL M147T, R148Q, and T154A Mutations

**Figure 5:**
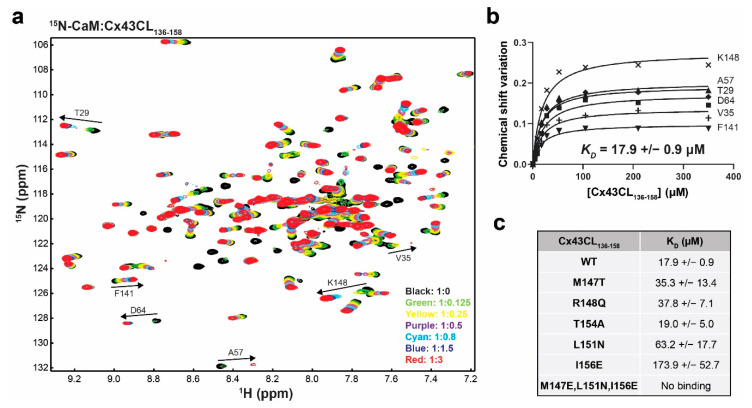
Impact of the Cx43CL ODDD mutations M147T, R148Q, and T154A on the interaction with CaM. (**a**) Example of an NMR titration experiment. ^15^N-HSQC spectrum of ^15^N-labeled CaM alone (black) has been overlaid with spectra collected in the presence of increasing molar ratios of unlabeled Cx43CL_136-158_ WT peptide (ratios indicated in the lower right corner). The residues used to calculate the dissociation constant (K_D_, panel **b**) have been labeled. (**b**) Example calculation of the K_D_. Representation of the binding isotherms for six residues. Final K_D_ and standard deviation were calculated by averaging the individual K_D_ obtained for each residue after plotting and linear fitting their ∆σ values. (**c**) Summary of the K_D_ obtained for the interaction of CaM with Cx43CL_136-158_ peptides containing various mutations.

Next, we addressed if these ODDD mutations would directly impact the ability of CaM to bind the Cx43CL. ^15^N-HSQC spectra of CaM were collected in the presence of various concentrations of Cx43CL peptides (K136-S158) containing the M147T, R148Q, or T154A ODDD mutations. Presented is the overlay of each spectrum after adding increasing molar ratio of the WT peptide and labeled are the residues used to calculate the dissociation constant (K_D_; Figure 5a,b). Of note, some of these residues are in intermediate exchange but peaks could be detected at a lower spectrum level permitting the K_D_ calculation. The K_D_ of 17.9 ± 0.9 μM between CaM and the Cx43CL WT peptide is similar to previously published values [17,29]. We attempted to solve the structure of the CaM and Cx43CL_136-158_ peptide complex. Unfortunately, after all the resonance assignments were collected, no amide chemical shifts were observed for CaM residues A1, D2, F19, M36, R37, L48, V55, E67, L69-M71, A73, K75-D78, D80-E83, F89, R90, F92, D93, L105-T110, L112, E114, and D122 and no α/β/other carbon chemical shifts were observed for M36, L69, M71, M72, R74-K77, T79, D80, E83, V91, F92, L105-N111, and E114 (Supplementary Figure S1 and Supplementary Table S1). The commonality among many of these residues, based upon structural analysis of CaM with a binding peptide from the Ca^+2^ pump [62], is they all would be involved in the direct interaction with the Cx43CL domain. These chemical shifts, which have broadened beyond detection, indicate the interaction is in intermediate exchange.

In comparison to the control, there was little-to-no difference in K_D_ between the M147T, R148Q, and T154A mutations (Figure 5c). The M147T result is particularly interesting because this is the predicted “1” position in the 1-5-10 motif. Therefore, to confirm the 1-5-10 motif described by Zhou et al. [17], we examined a series of additional mutations (Figure 5c). The position 5 mutant L151N and position 10 mutant I156E significantly reduced the K_D_ to 63.2 ± 17.7 μM and 173.9 ± 52.7 μM, respectively. The binding of CaM to the Cx43CL domain was lost when all three positions were mutated. Altogether, the data suggest that the ability of the Cx43CL to form α-helical structure and bind CaM do not contribute to the phenotype observed for the M147T, R148Q, and T154A mutations.

**Figure 6:**
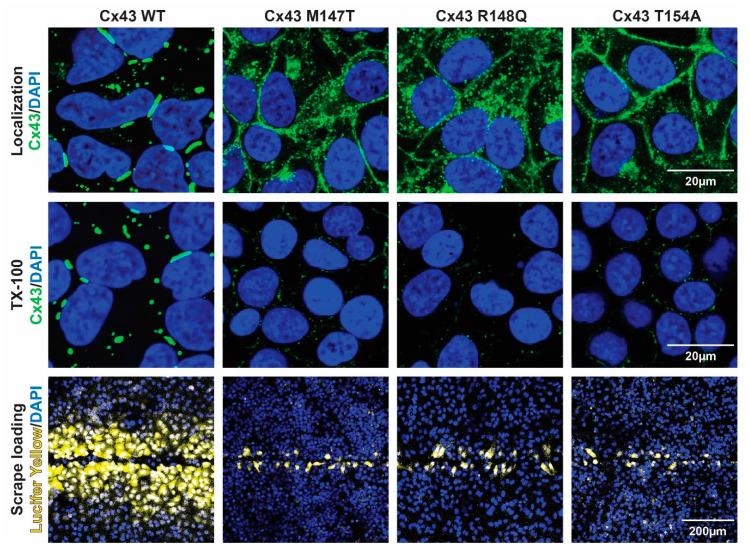
Effect of the Cx43CL ODDD mutations M147T, R148Q, and T154A on cellular localization and intercellular communication. Top panel: Immunofluorescence of HeLa cells stably transfected with Cx43 WT or ODDD mutants M147T, R148Q, and T154A (green, Cx43; blue, DAPI). Middle panel: Immunofluorescence of the same HeLa cells after in situ Triton X-100 extraction (green, Cx43; blue, DAPI). Bottom panel: Level of gap junction communication for each cell line was estimated using the scrape loading dye transfer assay (yellow, Lucifer yellow; blue, DAPI).

To further elucidate the molecular mechanism(s) associated with the phenotype of these three ODDD mutants, we utilized an exogenous expression system. Cx-deficient HeLa cells were generated to stably express each of the Cx43CL ODDD mutants. The cellular localization (immunofluorescence), the nature of gap junction channels formed (Triton X-100 solubility), and the level of intercellular communication (scrape loading) were assessed (Figure 6). Cx43 WT in HeLa cells was found predominately in large gap junction plaques at the plasma membrane with only a small amount of Cx43 washed away in the presence of Triton X-100 (i.e., junctional pool, Triton X-100 insoluble; non-junctional pool, Triton X-100 soluble). Additionally, they were extensively coupled with respect to Lucifer Yellow demonstrating greater than a fifth-order dye transfer (i.e., cell layers past scrape). When compared to WT, each of the ODDD mutants had an increase in the level of intracellular localization and the proteins that trafficked to the plasma membrane were localized throughout the entire plasma membrane. Triton X-100 in situ detergent extraction revealed that almost all of these membrane connexons were Triton X-100 soluble (i.e., non-junctional), similarly to the intracellular Cx43 ODDD mutants. The observation of the non-junctional Cx43 at the plasma membrane is consistent with the inability of the ODDD mutants to transfer Lucifer Yellow (Figure 6, bottom panel).

### 3.6. Interaction between the Cx43CT Domain and CaM

**Figure 7:**
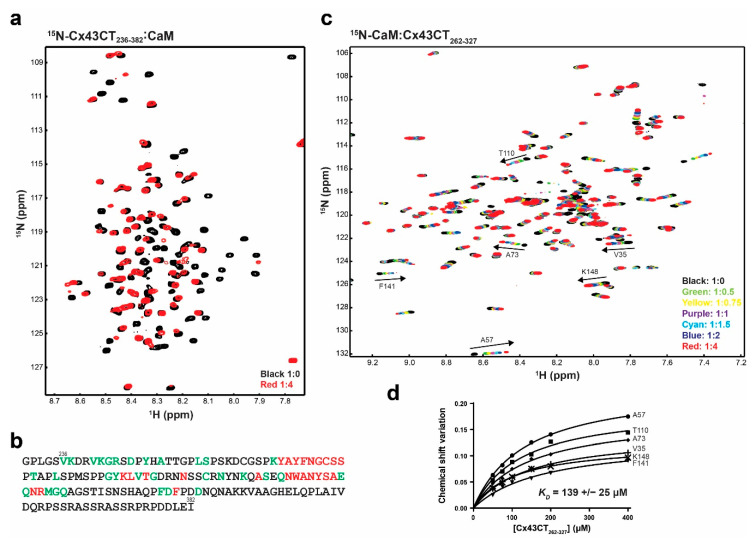
Characterizing the interaction between the Cx43CT_236-382_ domain and CaM. (**a**) ^15^N-HSQC spectrum of ^15^N-labeled Cx43CT_236-382_ alone (black) has been overlaid with the spectrum collected in the presence of unlabeled CaM in 10 mM CaCl_2_ (red, molar ratios indicated in the lower right corner). (**b**) Summary of the Cx43CT residues affected by the interaction with CaM when CaCl_2_ is present (green, peaks shift; red, peaks broaden beyond detection). (**c**) ^15^N-HSQC spectrum of ^15^N-labeled CaM alone (black) has been overlaid with spectra collected in the presence of increasing molar ratios of unlabeled Cx43CT_262-327_ (ratios indicated in the lower right corner). The residues used to calculate the dissociation constant (K_D_, panel **d**) have been labeled. (**d**) Fitting of the binding isotherms. Overall K_D_ and standard deviation were calculated by averaging the individual K_D_ obtained for each residue.

Zhou et al. [17] used a program that searched for putative CaM-binding sites and identified a sequence with a low predictive score that spans the 4th transmembrane-Cx43CT domain containing residues N224-Y247 [63]. Based upon the primary sequence, the expected start of the Cx43CT domain is residue V236. Thus the cytoplasmic exposed portion (residues V236-Y247) is long enough to bind CaM. To test this, we performed a NMR titration experiment (^15^N-HSQC) using the purified full-length ^15^N-labeled Cx43CT domain (residues V236-I382) and unlabeled CaM (Figure 7a, example using the Cx43CT/CaM 1:4 molar ratio spectrum). CaM affected most of the N-terminal portion of the Cx43CT domain between V236 and Q322 (Figure 7b). Unexpectedly, peaks that broadened beyond detection (red), indicating a stronger binding affinity than peaks that shifted (green), are localized between Cx43CT residues Y265 and R319, outside of the predicted V236-Y247 residues. This finding of CaM binding the Cx43CT is specific as the interaction is lost when Ca^+2^ is not present (Supplementary Figure S2). These results were confirmed when the data were collected from the ^15^N-CaM point of view (Supplementary Figure S3). A K_D_ of 139 ± 25 μM was calculated by holding the concentration of ^15^N-CaM constant and titrating with a polypeptide containing the Cx43CT residues affected by CaM (Figure 7c,d).

### 3.7. Impact of CaM Binding on the Phosphorylation of the Cx43CT Domain

**Figure 8:**
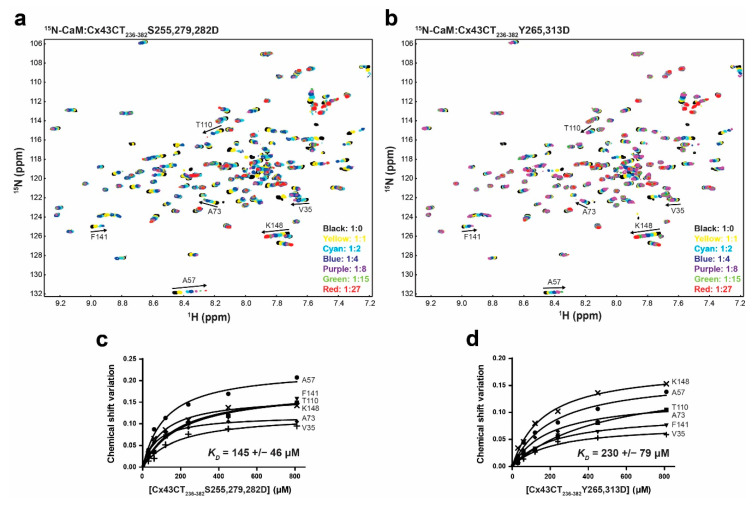
Effect of Cx43CT phosphorylation by MAPK and Pyk2/Src on the interaction with CaM. ^15^N-HSQC spectrum of ^15^N-labeled CaM alone (black) has been overlaid with spectra collected in the presence of increasing molar ratios of unlabeled Cx43CT_236-382_ with (**a**) MAPK S255,279,282D or (**b**) Pyk2/Src Y265,313D phosphomimetic mutants (ratios indicated in the lower right corner). The residues used to calculate the dissociation constant (K_D_, panel **c**,**d**) have been labeled. Changes in chemical shift (Δσ) plotted as a function of the Cx43CT_236-382_ (**c**) MAPK or (**d**) Src phosphomimetic mutants. Overall K_D_ and standard deviation were calculated by averaging the individual K_D_ obtained for each residue after plotting and linear fitting their Δσ values.

Studies have suggested that CaM binding the Cx43CT will not play a role in channel coupling [25,64,65]; however, the area affected by CaM is over Cx43CT residues phosphorylated by MAPK (ERK1 and ERK2; S279 and S282) and Pyk2/Src (Y265 and Y313). Therefore, we tested if the binding of CaM would be affected by or affect the phosphorylation of the Cx43CT domain. The same NMR titration experiment (^15^N-HSQC) was performed as described above for ^15^N-CaM, this time using two mutant versions of the Cx43CT with substitution of aspartic acids to mimic phosphorylation at those sites (MAPK, S255/279/282D; Pyk2/Src, Y265/313D). Upon addition of either the MAPK (Figure 8a) or Pyk2/Src (Figure 8b) Cx43CT phosphomimetics, a number of the ^15^N-CaM resonances peaks shifted and/or disappeared in a similar manner as Cx43CT WT (Supplemental Figure 3 and Figure 7c vs. Figure 8). Calculation of the binding affinity revealed that the MAPK (Figure 8c, K_D_ = 145 ± 46 μM) and Pyk2/Src (Figure 8d, K_D_ = 230 ± 79 μM) Cx43CT phosphomimetics had little-to-no effect on the binding of CaM for the Cx43CT (WT K_D_ = 139 ± 25 μM).

**Figure 9:**
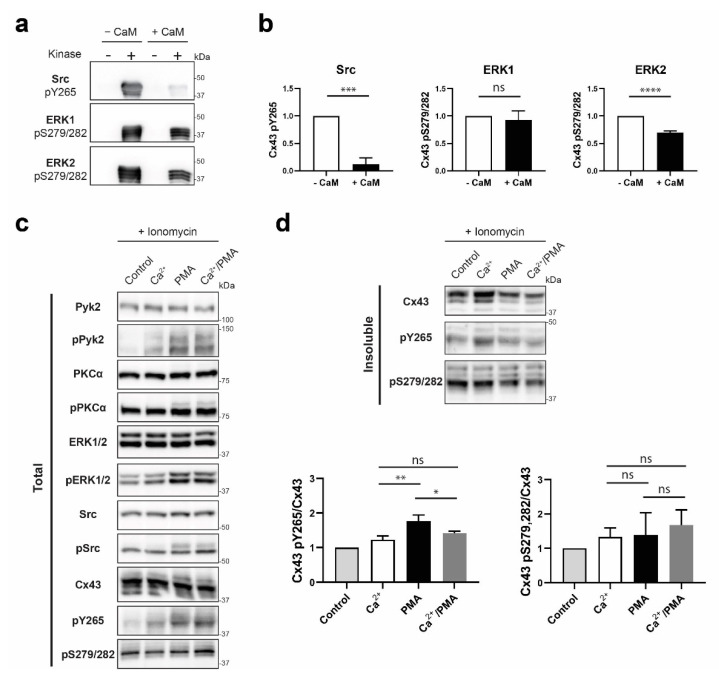
Determining if the CaM interaction with Cx43CT affects MAPK and Pyk2/Src phosphorylation of the Cx43CT domain. (**a**) Src, ERK1, or ERK2 were used in an in vitro kinase assay performed with purified GST-Cx43CT_236-382_ as substrate, in presence or absence of CaM. Phosphorylation was detected by Western blot using Cx43 Y265 or S279/S282 phospho-specific antibodies. (**b**) Quantification of the phosphorylation level from three independent experiments using Figures the iBright Analysis Software and analyzed in GraphPad Prism 8.0 (Student’s *t*-test, *** *p* < 0.0005, **** *p* < 0.0001). HeLa^Cx43^ cells were treated with ionomycin and Ca^2+^, or PMA, or Ca^2+^/PMA for 30 min. Lysates were then subjected to Triton X-100 extraction. Total lysate (**c**) and insoluble (**d**) fractions were analyzed by Western blot. Antibodies used are labeled on the left of each panel. Quantification of the Cx43 pY265 and pS279/282 phosphorylation levels were obtained from three independent experiments using the iBright Analysis Software and analyzed in GraphPad Prism 8.0 (Ordinary one way Anova, * *p* < 0.05, ** *p* < 0.01).

Finally, we addressed if CaM, once bound to the Cx43CT, would impact the phosphorylation of the Cx43CT by MAPK and Src (or Pyk2; [66]). An in vitro kinase assay was performed using the soluble Cx43CT_236-382_. In absence of CaM, an expected banding pattern typical of multiple phosphorylated species was observed (Figure 9a). In presence of CaM (~90% saturation), Src and ERK2 phosphorylation was inhibited by ~90% and ~30%, respectively (Figure 9b) while the effect on ERK1 phosphorylation was not statistically significant. Next, we determined if the loss of Cx43 phosphorylation by Src (or Pyk2) and ERK1 occurs in cyto. A Triton X-100 solubility assay was performed after treating HeLa^Cx43^ cells with ionomycin (Ca^2+^ ionophore) and Ca^2+^ (elevate intracellular Ca^2+^ concentration), or PMA (activates Pyk2, Src, and ERK1/2; Figure 9c), or Ca^2+^/PMA. PMA triggers Cx43 phosphorylation and subsequent internalization of the gap junctional pool. However, we sought to observe the effect of Ca^2+^/CaM on the phosphorylation levels of the Cx43 specifically localized at the membrane. To that effect, the insoluble fraction of a Triton X-100 extraction was analyzed (Figure 9d). As expected, PMA decreased the level of total Cx43 at the plaque while increasing Y265 phosphorylation. When the concentration of Ca^2+^ was elevated (increasing the CaM binding affinity for Cx43), there was no change in the level of pS279/282. However, the phosphorylation of Y265 in the junctional pool decreased by ~20%. Altogether, the in vitro and in cyto data are consistent with the ability of CaM to restrict Cx43 phosphorylation by Src (and/or Pyk2).

## 4. Discussion

pH-induced closure of Cx43 channels follows a “particle-receptor” model where the CL region containing residues D119-K144 was identified as the “receptor” for the CT domain acting as the gating “particle” [9,14,15]. Structural analysis using NMR showed that acidification induced α-helical order in the Cx43L2, increasing the affinity of the CT-CL interaction [14]. Here, we extended our structural study of the Cx43CL domain by characterizing a construct containing the full-length cytoplasmic loop (amino acids M100-Y155). Unexpectedly, unlike the Cx43L2, no α-helical structure could be modeled by NMR as NOE connectivities needed for secondary and tertiary structure were undetectable. Compared to the Cx43L2, the full-length CL construct includes 20 more residues on the N-terminus and 10 more on the C-terminus. We believe that the lack of α-helical structure in the full-length Cx43CL is a direct result of the addition of more intrinsically disordered residues, which translated into a more flexible protein and therefore pushed the α-helix/intrinsically disordered equilibrium toward the disorder state. This is consistent with the observation that three more residues added to the C-terminus (I156-S158) caused the Cx43CL to form more than one stable disordered conformation. We would like to point out, that in the context of the channel, we hypothesize that the α-helix/intrinsically disordered equilibrium would be pushed toward the ordered state because of the tethering to the second and third transmembrane domains. This possibility is supported by our studies that showed increased structural stability when the Cx43CT was tethered to its 4th transmembrane domain vs. the soluble version [67].

Another mechanism that leads to inhibition of gap junction intercellular communication is the Ca^2+^-dependent interaction of CaM with the Cx43CL domain (for review, see [28]). Zhou et al. [17] identified that CaM interacts with a Cx43CL peptide comprising residues K136-S158 and that its α-helical contents increases upon CaM binding. They also used a prediction program for CaM-binding sites [63], which identified that residue K136-S158 contains a 1-5-10 hydrophobic motif similar to other CaM binding proteins [68]. Our mutagenesis studies confirmed the predicted 1-5-10 hydrophobic motif and that all three sites need to be mutated in order to completely inhibit CaM from binding the Cx43CL. Additionally, we characterized three Cx43CL mutations within the CaM binding motif that cause ODDD (M147T, R148Q, and T154A; [37,38,39,40]) with the thought that the mechanism by which the disease manifests is either altered secondary structure or binding to CaM. The combination of CD and NMR revealed these Cx43CL mutants had the same propensity to form α-helical structure as well as bind CaM as the Cx43CL WT. The latter was surprising since all of the mutations are directly contained within the predicted 1-5-10 binding motif, and M147T, in particular, is located at the “1” position. However, using Cx-deficient HeLa cells stably transfected with the Cx43 ODDD mutants, we showed that the disease manifests most likely from an inability of Cx43 to traffic properly to the plasma membrane and/or form functional gap junction. This result was in agreement with several studies that used the Cx43 T154A mutant. Indeed, the Sosinsky lab identified T154 as being conserved among all Cxs, and discovered that its mutation to an alanine produced a “dominant negative channel-defective” mutant before it was even described as an ODDD causing mutation [39]. Consequently, further studies used this mutant as a tool to decipher if specific phenotypes were dependent or independent of the presence of a functional Cx43 channel [69,70]. Of note, and to the best of our knowledge, this study is the first one to address the structure and function of the M147T and R148Q mutants.

While the end point of decreased intercellular communication is similar, several laboratories have identified that in addition to the CL domain (e.g., Cx44 and Cx45 [18,20]), some connexins can actually interact with CaM through other and sometime multiple cytoplasmic domains (e.g., Cx35, Cx34.7, and Cx36 CT [71], Cx32 NT and CT [52,72,73], and Cx50 CL and CT [19,74]). In this study, we showed that this is also true for Cx43, as we discovered a direct interaction of CaM within an area of the Cx43CT-containing residues Y265-R319. Using the Calmodulation database and Meta-analysis predictor website (http://cam.umassmed.edu/ [75]), a potential 1-12 motif in the CT at positions L278 and V289 was detected, however this motif is not located within the most affected (i.e., tighter binding affinity) regions (Y265-S273 and N309-R319). Interestingly, the NMR solution structure of the Cx43CT revealed two areas with α-helical structure, one being within the residues affected by the interaction with CaM (A315-T326; [76]). Moreover, a study in our laboratory using a more native-like construct, Cx43CT attached to its fourth transmembrane domain, also revealed three short helical-like domains within this region [67]. These data suggest that this area has high potential for stable α-helical formation upon CaM binding as was observed for the Cx43CL and many other CaM binding partners [77].

Studies have shown that the Ca^2+^/CaM-dependent gating mechanism remains intact when Cx43 is truncated at CT residue M257 [27,64]. Therefore, what is the biological significance of this interaction? Upon examining the Cx43CT residues affected by the binding of CaM (Y265-R319), we noticed they directly overlap with the three areas of interaction by the developmentally regulated brain protein (Drebrin; residues K264-T275, S282-T290, and R299-G321; [78]). Drebrin functions as a linker between Cx43 gap junctions and the actin/submembrane cytoskeleton at the plasma membrane, and is required to maintain Cx43 gap junctions in their functional state [78,79]. We previously showed that phosphorylation at both Y265 and Y313 inhibits the Cx43 interaction with Drebrin [50]. Interestingly, these two tyrosine residues phosphorylated by Src/Pyk2 [66], as well as S279 and S282 phosphorylated by MAPK, are located within the CaM binding domain. Our data suggest that phosphorylation by Src/Pyk2 and MAPK would have no effect on the binding affinity of CaM for the Cx43CT. However, because prior binding of CaM was shown to inhibit subsequent phosphorylation of protein partners (for examples see, [80,81,82]), we sought to examine if the same mechanism exists for the Cx43CT. In an in vitro kinase assay, CaM inhibited Src phosphorylation of the Cx43CT by 90%, and to a lesser extent that of ERK2 (30%). Similarly, in presence of elevated intracellular Ca^2+^ concentration in HeLa^Cx43^ and treatment with PMA (activation of Pyk2/Src and ERK1/2), the phosphorylation level of the Y265 residue decreased compared to resting concentration of Ca^2+^.

**Figure 10:**
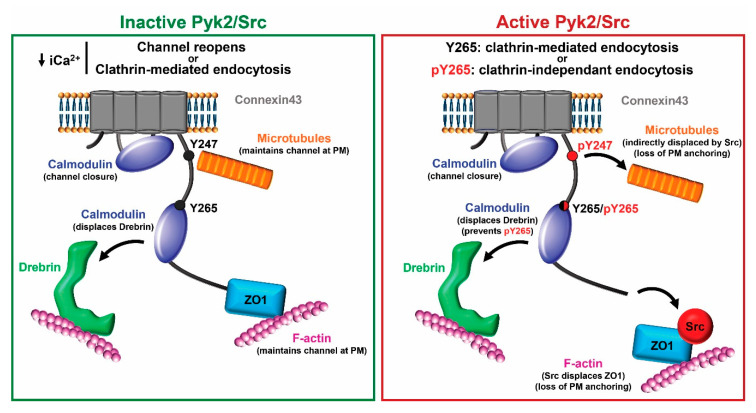
Proposed model for the role of CaM binding Cx43 with and without active Pyk2/Src. Left (inactive Pyk2/Src): Binding of CaM to the CL aids in closure of the channels by a structural change in the CL and/or steric hindrance of the pore. CaM also binds to the CT and displaces Drebrin, which interacts with F-actin. Once the level of intracellular Ca^2+^ decreases, the channels could reopen because Cx43 maintains its interaction with the microtubule and actin network (via ZO-1) or exposure of the AP-2 binding site Y_265_XXΦ upon CaM release would promote clathrin-mediated endocytosis. Right (active Pyk2/Src): Pky2/Src phosphorylation of Y247 displaces the microtubule and the direct interaction between Src and ZO-1 displaces ZO-1 as well as F-actin from Cx43. These channels are marked for internalization. However, the mode of internalization, clathrin-mediated or clathrin-independent, depends on the level of protection from phosphorylation at residue Y265 provided by CaM. Of note, this is a simplistic model that does not illustrate the many other protein partners that are involved in the regulation of Cx43. PM: plasma membrane.

Based upon these new data, we propose the following model for closure of Cx43 gap junction channels involving CaM and the level of Pyk2/Src activity (Figure 10). If Pyk2/Src is inactive, an increase in intracellular Ca^2+^ facilitates the binding of CaM to the CL, which in turn aids in the closure of the channels by a structural change in the CL (and its tethered transmembrane domains 2 and 3) and/or steric hindrance of the pore (for review, see [28,83]). CaM also binds to the CT and could displace Drebrin (tighter binding affinity, ~139 μM vs. ~300 μM). While CaM can be seen as mimicking Pyk2/Src phosphorylation to displace Drebrin and internalize Cx43, the major difference is that Y247 would not be phosphorylated by Pyk2/Src nor ZO-1 displaced by Src [57]. This would maintain the necessary interaction with the microtubule and actin network, respectively, allowing Cx43 to stay at the plasma membrane [84,85,86]. This could provide the chance for the channel to reopen once there is a decrease in the level of intracellular Ca^2+^ as seen in [25,27,64]. If internalization is to occur, the release of CaM would expose the AP-2 binding site Y_265_XXΦ to promote clathrin-mediated endocytosis [87]. If Pyk2/Src are active, our data suggests that CaM can limit Src phosphorylation of Cx43 residue Y265. It would be tempting to speculate that this is one mechanism by which clathrin-mediated endocytosis is favored over clathrin-independent endocytosis [88], since tyrosine phosphorylation of the Y_265_XXΦ motif would inhibit the binding of AP-2 (e.g., [89]).

## 5. Conclusions

Structural characterization of the full-length CL domain of Cx43 revealed an intrinsically disordered protein with propensity to form α-helical structure. The Cx43CL domain contains a CaM 1-5-10 binding motif in which ODDD disease causing mutations M147T, R148Q, and T154A did not affect the Cx43CL structure or interaction with CaM. Our data suggest these mutations manifest the disease from an inability of Cx43 to traffic to the plasma membrane and/or to form functional gap junctions. Unexpectedly, CaM directly interacted with the Cx43CT domain. While most likely not necessary for direct channel closure, the CaM interaction with the Cx43CT decreases Pyk2/Src phosphorylation of Y265. This could have potential implications in both regulating mechanisms of channel function and internalization based upon activity of other signaling pathways.

## Supplementary Materials

The following are available online at https://www.mdpi.com/2218-273X/10/10/1452/s1. **Figure S1**: ^15^N-HSQC of the CaM/Cx43CL_136-158_ complex. ^15^N-HSQC spectrum of ^15^N-labeled CaM in 100 mM KCl, 10 mM CaCl_2_ at pH 6.5, in presence of 1.2 molar equivalent of unlabeled Cx43CL_136-158_ peptide. Peaks present on the HSQC were assigned and have been labeled. **Figure S2**: Ca^2+^ dependency of the Cx43CT_236-382_/CaM interaction. ^15^N-HSQC spectrum of ^15^N-labeled Cx43CT_236-382_ alone (black) has been overlaid with the spectrum collected in the presence of unlabeled CaM and 10 mM EGTA (red, molar ratios indicated in the lower right corner). **Figure S3**: ^15^N-HSQCs of the CaM/Cx43CT_236-382_ complex. ^15^N-HSQC spectrum of ^15^N-labeled CaM alone (black) has been overlaid with spectra collected in the presence of unlabeled Cx43CT_236-382_ (red and green, molar ratios indicated in the lower right corner) in either (**a**) 10 mM CaCl_2_ or (**b**) 10 mM EGTA. Supplemental **Table S1**. Chemical shift assignments for the ^15^N, ^13^C-CaM/Cx43CT complex.
