# Supplementary material for: Calmodulin Directly Interacts with the Cx43 Carboxyl-Terminus and Cytoplasmic Loop Containing Three ODDD-Linked Mutants (M147T, R148Q, and T154A) that Retain α-Helical Structure, but Exhibit Loss-of-Function and Cellular Trafficking Defects
Source: Biomolecules. 2020 Oct 17;10(10):1452. doi: 10.3390/biom10101452 (PMC7602980; doi:10.3390/biom10101452)
Supplement: Supplementary file 1 [file biomolecules-10-01452-s001.pdf]

Supplemental Table I. Chemical shift assignments for the ^{15}N, ^{13}C-CaM/Cx43CT complex.

**Residue**	**$^{15}N(H^N)$**	**^{13}CO**	**$C_\alpha(H^\alpha)$**	**$C_\beta(H^\beta)$**	**Other Side Chain**
A1			51.77(3.99)	19.39(1.43)	
D2		175.64	54.72(4.60)	41.32(2.59,2.68)	
Q3	120.05(8.39)	175.69	55.21	29.77	
L4	123.36(8.34)	177.70	54.40(4.59)	43.49(1.41,1.66)	C^γ, 26.97(1.67); $C^{\delta1}$, 24.51(0.84); $C^{\delta2}$, 26.77(0.88)
T5	113.11(8.71)	175.43	60.37(4.37)	71.04(4.65)	$C^{\gamma2}$, 21.78(1.27)
E6	120.49(9.04)	179.51	60.07	29.19	
E7	119.52(8.72)	179.22	59.97	29.02	
Q8	120.45(7.72)	178.13	58.72	28.98	
I9	119.31(8.35)	177.76	66.24(3.56)	37.86(1.85)	$C^{\gamma1}$, 30.20(1.72,1.00); $C^{\gamma2}$, 17.33(1.04); $C^{\delta1}$, 12.98(0.77)
A10	121.13(8.00)	181.03	55.06(4.03)	17.76(1.44)	
E11	119.79(7.74)	180.32	59.11	29.12	
F12	120.08(8.63)	178.80	58.87		
K13	123.59(9.24)	179.23	60.07	31.75	
E14	120.35(7.84)	179.61	59.45	29.13	
A15	122.36(8.09)	178.96	55.40(4.31)	18.14(1.86)	
F16	118.8(8.7)	177.68	61.78(3.22)	39.57(3.08,3.31)	
S17	113.30(8.08)	174.56	61.43(4.00)	63.22(3.94,3.94)	
L18	121.05(7.29)		57.09		
F19		176.91	59.19	41.25	
D20	117.49(7.89)	177.25	52.46(4.47)	39.06(1.43,2.25)	
K21	124.40(7.76)	178.14	58.52	32.47	
D22	114.00(8.00)	177.68	52.70	39.39	
G23	109.37(7.67)	175.17	47.11(3.77,3.77)		
D24	120.83(8.45)	177.43	53.52	40.42	
G25	112.90(10.52)	173.79	45.38(4.28,3.63)		
T26	112.32(8.17)	173.06	59.57(5.34)	72.73(3.76)	$C^{\gamma2}$, 22.10(0.96)
I27	126.67(9.87)	176.02	60.88(4.78)	39.80(1.60)	$C^{\gamma1}$, 26.81(1.05,0.10); $C^{\gamma2}$, 17.46(0.77); $C^{\delta1}$, 15.34(0.16)
T28	116.43(8.37)	176.67	59.31(4.74)	72.46(4.74)	$C^{\gamma2}$, 21.71(1.24)
T29	112.43(9.26)	177.39	66.30(3.71)	67.77(4.14)	$C^{\gamma2}$, 23.26(1.20)
K30	121.27(7.61)	179.91	59.17	32.41	
E31	122.08(7.78)	179.38	59.32	32.35?	
L32	120.08(8.63)	179.03	58.36	42.62	
G33	105.74(8.75)	175.16	48.20(3.90,3.52)		
T34	118.57(8.13)	177.20	67.04(3.86)	68.76(4.27)	$C^{\gamma2}$, 21.34(1.19)
V35E31	121.85(7.52)	179.58	66.65(3.38)		$C^{\gamma1}$, 20.39(0.45); $C^{\gamma2}$, 22.63(0.60);

M36					
R37		181.44	59.11	29.92	
S38	119.17(7.94)	175.02	61.63(4.30)	62.63(4.01,3.95)	
L39	120.34(7.38)	177.10	54.76	42.04	
G40	106.34(7.86)	174.35	45.41		
Q41	118.19(7.78)	174.21	54.25	30.31	
N42	116.40(8.77)	171.92	51.23	39.24	
P43		177.77	62.36(4.68)	31.80(2.15,1.85)	C^{γ}, 29.73(2.00,1.89); C^{δ}, 49.86(3.55,3.18)
T44	113.15(8.80)	175.16	60.38	70.95	
E45	120.74(8.82)	178.96	59.93	28.86	
A46	120.81(8.28)	180.22	55.06(4.03)	18.11(1.31)	
E47	118.74(7.71)	180.22?	59.07	29.55	
L48		178.64	57.78	42.48	
Q49	118.54(8.30)	178.52	58.48	28.12	
D50	120.44(8.11)	178.75	57.67	40.15	
M51	119.30(7.79)	179.05	59.27	33.43	
I52	118.12(8.00)	178.12	64.06(3.48)	36.87(1.93)	$C^{\gamma1}$, 28.08(1.47,1.22); $C^{\gamma2}$, 16.17(0.63); $C^{\delta1}$, 12.13(0.62)
N53	118.40(8.70)	177.28	55.72	37.97	
E54	116.13(7.45)	177.29	58.74	30.28	
V55		175.65	60.66	32.99	
D56	121.15(7.75)	175.91	53.77	40.68	
A57	131.66(8.29)	178.69	54.16(4.15)	19.66(1.46)	
D58	114.15(8.22)	177.78	52.81	39.71	
G59	108.69(7.61)	174.97	47.09(3.81,3.71)		
N60	118.75(8.16)	176.87	52.59	37.55	
G61	113.30(10.54)	173.28	45.58(4.17,3.41)		
T62	108.61(7.65)	173.26	59.41	72.25(3.94)	$C^{\gamma2}$, 22.52(1.05)
I63	123.29(8.74)	175.61	59.90(5.06)	40.04(1.93)	$C^{\gamma1}$, . (1.50,1.18); $C^{\gamma2}$, 18.51(1.19); $C^{\delta1}$, 13.64(0.74)
D64	128.33(8.94)	176.21	51.99(5.40)	42.07(2.77,3.04)	
F65	118.78(9.00)	173.63	63.25	36.11	
P66			66.72(3.82)	30.67(2.16,1.89)	C^{γ}, .(.,.); C^{δ}, 49.02(3.69,3.69)
E67		179.33	59.14		
F68	123.99(8.80)		61.48		
L69					
T70			66.30(3.71)	68.47(4.18)	$C^{\gamma2}$, 21.78(1.13)
M71					
M72	117.4(7.95)				

A73			55.08(3.97)	18.26(1.35)	
R74	116.5(7.5)				
K75					
M76					
K77					
D78		176.67	54.37(4.62)	41.32(2.59,2.68)	
T79	114.05(7.99)				
D80					
S81			58.73(4.39)	63.75(3.97,3.89)	
E82		178.52	59.34	29.60	
E83					
E84	118.5(8.1)	178.94	59.16	29.10	
I85	121.73(8.08)	177.96	65.19(3.86)	37.32(2.09)	$C^{\gamma 1}$, 30.02(1.77,1.77); $C^{\gamma 2}$, 19.06(1.02); $C^{\delta 1}$, 13.30(0.70)
R86	121.74(8.43)		60.02	29.79	
E87	119(8.35)	179.86	59.16	29.10	
A88	121.73(8.08)		55.29(4.05)	18.05(1.68)	
F89			62.25(3.02)	38.90(2.92,3.14)	
R90		177.78			
V91	117.61(7.41)				
F92					
D93		177.47	52.68(4.51)	39.06(1.43,2.25)	
K94	125.89(7.62)	178.18	59.26	32.90	
D95	113.93(8.21)	177.78	52.81	39.71	
G96	109.56(7.85)	175.13	47.11(3.77,3.77)		
N97	119.78(8.33)	176.05	52.64	37.92	
G98	113.18(10.72)	172.45	44.97(3.97,3.34)		
Y99	116.06(7.61)	174.52	55.99	43.08)	
I100	127.38(10.15)	175.51	60.69(4.71)	38.87(1.76)	$C^{\gamma 1}$, 26.83(1.16,0.15); $C^{\gamma 2}$, 17.60(0.89); $C^{\delta 1}$, 15.96(0.22)
S101	123.89(8.95)	175.25	55.70(4.78)	66.56(4.36,3.90)	
A102	123.13(9.25)	179.29	55.93(3.84)	17.91(1.40)	
A103	118.35(8.27)	181.52	54.82(3.97)	18.00(1.35)	
E104	120.33(7.92)		59.58		
L105					
R106					
H107					
V108					
M109					

T110					
N111	121.15(7.75)				
L112		176.42			
G113	106.17(7.69)	174.33	45.24(4.19,3.71)		
E114					
K115	123.5(8.6)	175.72	56.14		
L116	125.52(8.15)	117.97	53.76	45.10	
T117	114.77(9.27)	175.44	60.37(4.37)	71.04(4.65)	$C^{\gamma 2}$, 21.71(1.24)
D118	121.10(8.92)	178.61	57.99	39.65	
E119	119.10(8.66)	179.20	59.95	28.98	
E120	120.53(7.78)	179.90	59.27	30.66	
V121	120.18(8.10)		67.01(3.48)	31.45(2.15)	$C^{\gamma 1}$, 22.10(0.96); $C^{\gamma 2}$, 23.93(0.89);
D122		179.18	57.57	40.60	
E123	119.47(8.06)	178.79	59.14	29.45	
M124	120.3(8.3)	179.13	59.20	33.60	
I125	118.15(7.71)	177.15	63.83(3.40)	36.17(2.04)	$C^{\gamma 1}$, 28.08(1.47,1.22); $C^{\gamma 2}$, 16.17(0.63); $C^{\delta 1}$, 10.69(0.63)
R126	118.16(8.20)	179.33	59.65	30.10	
E127	116.08(7.93)	177.31	58.56	29.78	
A128	118.77(7.39)	177.97	52.07(4.34)	21.17(1.35)	
D129	117.47(7.93)	176.04	54.02	40.34	
I130	127.92(8.39)	177.87	63.25(3.83)	38.51(1.92)	$C^{\gamma 1}$, 27.86(1.61,1.25); $C^{\gamma 2}$, 17.26(0.87); $C^{\delta 1}$, 12.18(0.81)
D131	116.60(8.28)	178.27	53.69	39.87	
G132	108.57(7.57)	175.30	47.51(3.90,3.74)		
D133	120.84(8.32)	177.59	53.52	40.23	
G134	113.02(10.39)	172.82	45.67(3.95,3.34)		
Q135	115.39(7.97)	174.69	53.07	32.16	
V136	125.41(9.14)	175.84	61.68(5.11)	33.60(2.21)	$C^{\gamma 1}$, 21.85(1.20); $C^{\gamma 2}$, 22.30(0.81);
N137	129.17(9.52)	174.90	51.23	38.13	
Y138	118.55(8.45)	176.08	62.72	37.64	
E139	118.57(8.13)	180.60	60.29	28.81	
E140	119.83(8.77)	179.41	58.38	29.75	
F141	124.78(8.90)	176.80	61.59	39.92	
V142	119.42(8.58)	179.60	67.06(3.03)	31.58(1.78)	$C^{\gamma 1}$, 21.20(0.66); $C^{\gamma 2}$, 22.90(0.42);
Q143	118.36(7.47)	177.93	58.79	28.01	
M144	119.07(7.86)	177.87	58.41	32.99	
M145	114.03(7.74)	177.04	55.36	32.36	
T146	110.60(7.53)	174.16	62.14(4.23)	70.34(4.15)	$C^{\gamma 2}$, 21.28(1.08)

A147	127.35(7.81)	176.68	52.84(4.20)	19.08(1.31)	
K148	126.42(7.97)	181.45	57.50	33.74	
